# Hospital Based‐Exercise Interventions for persons living with Alzheimer’s Disease and Related Dementias

**DOI:** 10.1002/alz.091953

**Published:** 2025-01-09

**Authors:** Elizabeth Reaves Houston, David H Lynch

**Affiliations:** ^1^ UNC Chapel Hill, Chapel Hill, NC USA

## Abstract

**Introduction:**

Among persons living with Alzheimer’s Disease and Related Dementias (PwD), ∼25% are hospitalized yearly, during which >95% of their time is spent in bed, leading to functional decline for 50% of hospitalized PwD. Hospital‐based exercise interventions are a proposed strategy to mitigate this risk of hospital‐associated functional decline. However, the applicability of hospital‐based exercise intervention trials for PwD is poorly understood. Our narrative review explores the inclusion of PwD within such trials and the effectiveness of hospital‐based exercise interventions in mitigating functional decline among PwD.

**Methods:**

This review follows PRISMA‐Scr guidelines. The CENTRAL and MEDLINE databases and ClinicalTrials.gov were searched to identify randomized, controlled trials of hospital‐based exercise interventions for older adults (≥65). Eligibility criteria were defined a priori. Studies were screened at the abstract and full‐text level by one reviewer. A pre‐defined abstraction form was used to identify study characteristics, including details of the exercise intervention, functional outcomes, and adverse events. When reported, we abstracted eligibility criteria relevant to cognitive status and proportion of PwD among the study population.

**Results:**

Of 882 articles screened, 27 met the eligibility criteria. Seventeen trials included PwD: 9 with a hospital‐based exercise intervention and 8 with a multi‐component or unit‐based intervention. Four studies of hospital‐based exercise interventions and 3 studies of multi‐component interventions reported a proportion of PwD. Of the 9 trials studying an exercise intervention, 5 found a non‐significant change in functional capacity between admission and discharge within the intervention group, and 4 found a significant difference in functional capacity. No studies stratified outcomes by the presence of dementia or cognitive impairment.

**Conclusion:**

PwD are eligible for most trials of hospital‐based exercise interventions for older adults included in this review. However, we cannot determine the applicability of hospital‐based exercise interventions for PwD due to the inconsistency in reporting the prevalence of dementia within each study population. Further research must facilitate our ability to apply the results of hospital‐based exercise interventions to PwD, most directly by designing and testing hospital‐based exercise interventions specifically for PwD.

